# JAK2 in pediatric leukemia: mechanisms of pathogenesis and drug development – a narrative review

**DOI:** 10.1097/MS9.0000000000003180

**Published:** 2025-03-18

**Authors:** Emmanuel Ifeanyi Obeagu

**Affiliations:** Department of Biomedical and Laboratory Science, Africa University, Mutare, Zimbabwe

**Keywords:** drug development, JAK2, pathogenesis, pediatric leukemia, targeted therapy

## Abstract

Pediatric leukemia is one of the most prevalent childhood malignancies, and its pathogenesis involves complex molecular and genetic mechanisms. Among the critical players in leukemogenesis, the Janus kinase 2 (JAK2) gene has garnered significant attention due to its role in aberrant signal transduction and hematopoietic dysregulation. Mutations such as JAK2 V617F, while more commonly associated with myeloproliferative neoplasms, have also been implicated in pediatric leukemia subtypes, including acute lymphoblastic leukemia and acute myeloid leukemia. These mutations result in constitutive activation of the JAK-STAT pathway, promoting unchecked cell proliferation, survival, and resistance to apoptosis. The dysregulation of the JAK2 signaling pathway not only drives malignant transformation but also facilitates interactions with the bone marrow microenvironment, creating a niche that supports leukemia cell survival and immune evasion. Therapeutic advancements have focused on JAK2 inhibitors such as ruxolitinib and fedratinib, which show promise in preclinical and early clinical settings. However, challenges such as drug resistance and off-target effects limit their efficacy, necessitating the exploration of combination therapies and novel drug formulations. Current strategies include combining JAK2 inhibitors with chemotherapy, immune checkpoint inhibitors, or epigenetic modulators to achieve synergistic effects.

## Introduction

Pediatric leukemia remains one of the most commonE and challenging types of childhood cancers, accounting for approximately 30% of all pediatric malignancies. The majority of cases are acute lymphoblastic leukemia (ALL), followed by acute myeloid leukemia (AML). Despite significant advances in treatment and survival rates over the past few decades, leukemia continues to be a leading cause of cancer-related deaths in children. The complexities involved in leukemia pathogenesis, including the roles of genetic mutations, aberrant signaling pathways, and the tumor microenvironment, contribute to the difficulty in treating this disease effectively. One such critical genetic factor that has emerged as a potential driver of pediatric leukemia is the Janus kinase 2 (JAK2) gene^[1,2]^. JAK2 is a non-receptor tyrosine kinase that plays a pivotal role in cytokine and growth factor signaling, essential for normal hematopoiesis. It is involved in transmitting signals from cell surface receptors to the nucleus through the activation of the JAK-STAT pathway. The JAK-STAT signaling cascade is critical for controlling various cellular processes, including cell proliferation, survival, differentiation, and immune response regulation. Any mutations that lead to the dysregulation of JAK2 can have profound effects on these processes, contributing to the development of malignancies, including leukemia. Although JAK2 mutations are commonly associated with adult hematological cancers like myeloproliferative neoplasms, there is growing evidence that JAK2 also plays a role in pediatric leukemia, including ALL and AML^[3,4]^. The pathogenesis of pediatric leukemia is multifactorial, involving both genetic and environmental factors. In particular, mutations in key oncogenes, tumor suppressor genes, and signaling pathways have been identified as driving the progression of leukemia in children. JAK2 mutations, specifically the JAK2 V617F mutation, have been widely studied in the context of adult cancers, especially myeloproliferative disorders.^[5,6]^

**HIGHLIGHTS**

- Explores JAK2 mutations driving aberrant signaling pathways in pediatric leukemia, leading to uncontrolled proliferation and survival of leukemic cells.
- Highlights JAK-STAT pathway activation as a key mechanism underlying pediatric leukemia progression and therapeutic resistance.
- Emphasizes JAK2 as a critical biomarker for early diagnosis and risk stratification in pediatric leukemia.
- Discusses advancements in JAK2 inhibitors, their efficacy, and challenges in pediatric leukemia treatment development.
- Reviews mechanisms of resistance to JAK2-targeted therapies and potential combination strategies to overcome treatment hurdles.

In pediatric leukemia, JAK2 mutations can lead to the constitutive activation of the JAK-STAT signaling pathway, a process that bypasses the normal regulation by cytokine receptors. This uncontrolled signaling pathway promotes leukemia cell proliferation, survival, and resistance to apoptosis, hallmarks of cancer cell behavior. Additionally, JAK2 mutations can alter the hematopoietic microenvironment, making it more supportive of leukemia cell growth. This disruption of normal hematopoiesis contributes to the progression of leukemia and complicates treatment efforts. The growing understanding of JAK2’s role in leukemia pathogenesis has important implications for the development of novel therapeutic approaches^[7,8]^. One of the primary challenges in treating pediatric leukemia is the genetic heterogeneity of the disease. Pediatric leukemia is not a single entity but a complex group of disorders with diverse genetic mutations and molecular profiles. JAK2 mutations are often found in conjunction with other genetic aberrations, which complicates the development of targeted therapies. In particular, the presence of JAK2 mutations in pediatric leukemia cells often confers resistance to traditional chemotherapy, leading to relapse and treatment failure.^[9,10]^ Over the past few years, targeted therapies aimed at inhibiting the JAK-STAT pathway have shown promise in treating various cancers, including hematological malignancies. JAK2 inhibitors, such as ruxolitinib and fedratinib, have been approved for use in adults with myeloproliferative disorders, and their potential in pediatric leukemia is actively being explored. These inhibitors work by blocking the JAK2 kinase activity, preventing the activation of downstream signaling pathways that promote cancer cell growth.^[11,12]^

In addition to monotherapy, combination therapies are being investigated to enhance the efficacy of JAK2 inhibitors in pediatric leukemia. Combining JAK2 inhibitors with chemotherapy or other targeted therapies, such as immune checkpoint inhibitors or epigenetic modulators, could help overcome the challenges posed by resistance mechanisms and improve therapeutic outcomes. This approach has the potential to achieve synergistic effects, enhancing the anti-leukemic activity while minimizing side effects.^[13-15]^ The tumor microenvironment (TME) also plays a crucial role in the progression of pediatric leukemia. Leukemia cells do not exist in isolation but interact with their surrounding environment, including stromal cells, immune cells, and the extracellular matrix. These interactions can enhance the survival and proliferation of leukemia cells, creating a supportive niche that shields them from chemotherapy and immune surveillance. JAK2 mutations may influence the TME by altering the production of cytokines, growth factors, and immune modulators, which in turn promote leukemia cell survival and immune evasion.^[16-18]^ Immune evasion is another critical aspect of JAK2-driven leukemia. JAK2 mutations can activate STAT3 and STAT5, transcription factors that promote the expression of immune checkpoint molecules, such as PD-L1, on leukemia cells. This immune evasion mechanism allows leukemia cells to escape recognition and destruction by T cells and natural killer cells.^[19-21]^

## Aim

The aim of this review is to provide a comprehensive analysis of the role of JAK2 in the pathogenesis of pediatric leukemia, exploring the underlying molecular mechanisms, the impact of JAK2 mutations, and the dysregulation of the JAK-STAT signaling pathway.

## Justification of the review

Pediatric leukemia remains one of the most common and challenging malignancies in children, with treatment strategies often associated with significant long-term side effects and variable success rates. Despite advances in conventional therapies, there is a critical need for targeted approaches that can improve outcomes and reduce toxicities, particularly for pediatric patients. The JAK2 gene, involved in the JAK-STAT signaling pathway, has emerged as a central player in the pathogenesis of various hematologic malignancies, including leukemia. JAK2 mutations have been implicated in leukemogenesis, contributing to uncontrolled cell proliferation, survival, and differentiation. While JAK2 inhibitors have shown promise in treating other malignancies, their application in pediatric leukemia has yet to be fully explored. This review is justified by the need to bridge the gap between existing preclinical and clinical data, focusing on the mechanisms of JAK2 involvement in pediatric leukemia and evaluating the potential of JAK2 inhibitors as targeted therapies for children. The review will also address the challenges associated with these therapies, such as drug resistance, microenvironmental interactions, and the specific pharmacokinetics required for pediatric populations. Furthermore, the integration of combination therapies, involving JAK2 inhibitors and other therapeutic modalities, is a growing area of interest that requires in-depth exploration to optimize treatment outcomes. This review serves to not only synthesize the current understanding of JAK2 in pediatric leukemia but also to provide a foundation for future research directions.^[22-24]^

## Review methods

This narrative review was conducted by systematically examining peer-reviewed articles, clinical studies, and scientific reports on JAK2 mutations in pediatric leukemia. The literature search was performed using databases such as PubMed, Google Scholar, and Scopus, focusing on publications from the last two decades to ensure the inclusion of the most relevant and recent findings. Key search terms included “JAK2 mutations in leukemia,” “pediatric leukemia and JAK-STAT signaling,” “JAK2 inhibitors,” and “targeted therapies in childhood leukemia.” Studies discussing the molecular mechanisms, drug development, clinical trials, and resistance mechanisms of JAK2-targeted therapies were prioritized. Both preclinical and clinical studies were reviewed to provide a comprehensive understanding of JAK2-mediated leukemogenesis, therapeutic strategies, and associated challenges. Due to the limited availability of pediatric-specific trials, findings from adult leukemia and myeloproliferative neoplasms (MPNs) were also considered, with caution in extrapolating results to pediatric cases. The review methodology was qualitative, focusing on thematic analysis of studies rather than statistical meta-analysis. Clinical trial data, where available, were referenced to assess the efficacy and safety of JAK2 inhibitors in pediatric patients. Limitations, gaps in research, and future directions were also discussed based on the identified literature. This review aims to provide a balanced, comprehensive, and up-to-date perspective on JAK2 in pediatric leukemia, emphasizing its role in disease progression and targeted drug development.

## Limitations of the review

While this narrative review provides a comprehensive analysis of JAK2 in pediatric leukemia, several limitations must be acknowledged:

### Lack of extensive clinical data

The review relies primarily on preclinical studies, adult leukemia data, and small-scale pediatric studies due to limited clinical trials involving JAK2 inhibitors in pediatric leukemia. Many findings are extrapolated from adult MPNs or acute leukemia, which may not fully reflect pediatric-specific disease mechanisms.

### Potential publication bias

The reviewed literature predominantly includes published, peer-reviewed studies, which may introduce publication bias by overlooking negative or inconclusive results that are less likely to be published. Emerging therapies and experimental data from ongoing clinical trials may not yet be publicly available, limiting the scope of recent advancements.

### Limited discussion on rare JAK2 variants

Most research has focused on the JAK2 V617F mutation, while other rare JAK2 mutations that may play a role in pediatric leukemia are not as well studied or discussed in detail. The complexity of co-occurring genetic mutations (e.g., CRLF2, PAX5, and IKZF1 alterations) and their interplay with JAK2 signaling requires further investigation beyond the scope of this review.

### Challenges in treatment comparisons

Due to variations in treatment regimens, patient populations, and study methodologies, direct comparisons between different JAK2-targeted therapies, chemotherapies, and immunotherapies are difficult. The review does not include meta-analyses or systematic reviews, which could provide more quantitative comparisons of treatment efficacy.

### Limited discussion on long-term outcomes

The long-term safety and efficacy of JAK2 inhibitors in pediatric leukemia remain uncertain, as most clinical data are based on short-term studies or adult populations. There is a lack of long-term follow-up data on disease relapse, late toxicities, and survival outcomes in pediatric patients treated with JAK2-targeted therapies.

### Emerging therapies and future directions

The review provides an overview of current drug development efforts, but rapid advancements in gene editing (e.g., CRISPR), novel drug formulations, and combination therapies may quickly outdate some conclusions. More in vivo studies, clinical trials, and real-world data are needed to validate the findings discussed in this review.

## Mechanisms of pathogenesis in JAK2-driven pediatric Leukemia

### JAK2 mutations in pediatric leukemia

JAK2 mutations have emerged as significant contributors to the pathogenesis of pediatric leukemia, a malignancy characterized by the uncontrolled proliferation of immature blood cells. The JAK2 gene encodes a non-receptor tyrosine kinase that is integral to the signaling pathways governing hematopoiesis and immune regulation. In healthy cells, JAK2 is activated by cytokine receptors, leading to the phosphorylation of downstream signaling molecules such as STAT (Signal Transducer and Activator of Transcription) proteins. This cascade promotes normal cell survival, proliferation, and differentiation. However, mutations in the JAK2 gene can lead to its constitutive activation, bypassing the need for cytokine signaling and contributing to the unchecked growth and survival of leukemia cells. These mutations have been identified in various hematologic cancers, including both adult and pediatric forms of leukemia^[4,25]^. The most well-known mutation in JAK2 is the JAK2 V617F mutation, a point mutation that results in the substitution of valine with phenylalanine at position 617. This mutation leads to the constitutive activation of JAK2, even in the absence of cytokines or growth factors. Although JAK2 V617F mutations are more commonly associated with myeloproliferative disorders such as polycythemia vera and essential thrombocythemia in adults, its role in pediatric leukemia has been increasingly recognized. In pediatric leukemia, the presence of JAK2 mutations, particularly the V617F mutation, is associated with a variety of outcomes, including increased leukemia cell proliferation and resistance to apoptosis. These mutations may be involved in both de novo leukemogenesis and the progression of pre-existing hematologic disorders in children^[26]^.

In addition to the JAK2 V617F mutation, other genetic alterations affecting JAK2 has been identified in pediatric leukemia, including deletions, amplifications, and other point mutations. These mutations often result in dysregulated JAK2 signaling that contributes to leukemia cell survival and proliferation. For example, mutations that lead to the upregulation of JAK2 expression or altered subcellular localization of the kinase have been observed in both AML and ALL in pediatric patients. The activation of the JAK-STAT pathway in these cases results in the activation of downstream transcription factors such as STAT5, which promote the transcription of genes involved in cell survival, proliferation, and self-renewal. As a result, these mutations contribute to leukemia progression and chemoresistance, highlighting the importance of JAK2 in the pathogenesis of pediatric leukemia^[27]^. In pediatric patients, JAK2 mutations may occur as part of complex genetic landscapes involving additional co-occurring mutations or chromosomal abnormalities. In AML, for instance, JAK2 mutations can be found alongside mutations in other genes like FLT3, CEBPA, or NPM1, which further complicate the disease and treatment response. Similarly, in ALL, JAK2 mutations are sometimes present alongside genetic alterations such as Philadelphia chromosome translocations or mutations in the BCR-ABL1 fusion gene, which are known to drive leukemogenesis. These complex mutational profiles underscore the need for comprehensive genetic screening in pediatric leukemia to accurately characterize the genetic drivers of the disease and tailor treatment strategies^[7]^.

**Figure 1.**
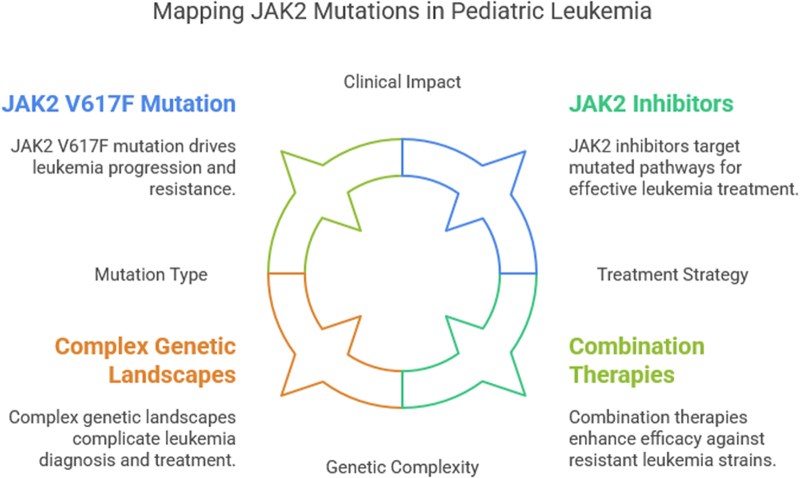
Mapping JAK2 mutations in pediatric leukemia.

The presence of JAK2 mutations in pediatric leukemia is not only a marker of disease progression but also a potential therapeutic target. The aberrant activation of JAK2 leads to the dysregulation of immune responses, further complicating leukemia treatment. For example, mutations in JAK2 may contribute to immune evasion by promoting the expression of immune checkpoint molecules like PD-L1, which inhibit T-cell activation and allow leukemia cells to escape immune surveillance. This immune escape is a significant challenge in the treatment of leukemia, and JAK2 mutations may help leukemia cells avoid the cytotoxic effects of chemotherapy and immunotherapy. Consequently, understanding the role of JAK2 mutations in immune regulation is critical for developing new therapeutic approaches that target both the leukemia cells and the immune evasion mechanisms they employ^[28]^. The identification of JAK2 mutations in pediatric leukemia patients has led to the exploration of targeted therapies that can directly inhibit JAK2 activity. JAK2 inhibitors, such as ruxolitinib, have shown efficacy in adult hematologic malignancies and are being evaluated in pediatric settings. These inhibitors work by blocking the kinase activity of JAK2, preventing its downstream signaling and inhibiting leukemia cell proliferation. In pediatric leukemia models, JAK2 inhibitors have demonstrated the ability to reduce leukemia cell growth and improve survival rates. However, the safety and pharmacokinetics of these drugs in children need to be further studied, as pediatric patients may respond differently than adults due to age-related differences in drug metabolism and organ function^[1,29]^. Furthermore, combination therapies that incorporate JAK2 inhibitors with chemotherapy or immune modulators are being explored to overcome the challenges of drug resistance and improve treatment outcomes. The combination of JAK2 inhibition with immune checkpoint inhibitors could help restore immune surveillance in patients with JAK2-driven leukemia, making these cells more susceptible to immune-mediated destruction (Fig. 1).^[30]^

### Dysregulation of the JAK-STAT pathway in pediatric leukemia

The JAK-STAT pathway plays a critical role in regulating hematopoiesis, immune function, and cellular proliferation by transducing extracellular signals from cytokines and growth factors into the cell. This signaling cascade is typically activated when cytokines bind to their respective receptors, which then recruit JAKs to the receptor complex. JAKs, in turn, phosphorylate the receptor, creating docking sites for STAT (Signal Transducer and Activator of Transcription) proteins. Once recruited, STAT proteins are phosphorylated by JAKs, dimerize, and translocate to the nucleus, where they regulate gene expression involved in cell survival, differentiation, and proliferation. In hematopoietic cells, this pathway is essential for the proper development and function of blood cells. However, mutations or alterations in the JAK-STAT pathway can lead to dysregulation, resulting in malignant transformations, including leukemia^[31]^. In pediatric leukemia, the dysregulation of the JAK-STAT pathway is a central event in leukemogenesis, contributing to uncontrolled cell growth and survival. This aberrant activation can occur through various mechanisms, such as mutations in the genes encoding JAKs or STATs, alterations in the receptors that activate the pathway, or upregulation of downstream signaling molecules. One of the most notable mutations in this pathway is the JAK2 V617F mutation, which causes constitutive activation of JAK2 kinase, bypassing the normal requirement for cytokine binding. This mutation leads to persistent signaling through the JAK-STAT pathway, driving leukemia cell proliferation and preventing apoptosis. Other mutations in JAK2 or STAT proteins may also lead to similar outcomes, including increased sensitivity to cytokines and dysregulated immune responses, which contribute to the malignant phenotype in pediatric leukemia^[32]^.

The dysregulation of JAK-STAT signaling in leukemia does not solely impact the leukemia cells but also disrupts the bone marrow microenvironment, further promoting disease progression. In leukemia, the TME, which includes stromal cells, immune cells, and extracellular matrix components, is often altered by aberrant JAK-STAT signaling. Leukemia cells can exploit this dysregulation to create a supportive niche that promotes their survival and evades immune surveillance. For example, JAK2 mutations in leukemia cells can lead to the production of cytokines and growth factors such as IL-6, IL-10, and GM-CSF, which not only stimulate leukemia cell proliferation but also recruit immunosuppressive cells, like regulatory T-cells (Tregs), to the tumor microenvironment. This creates a feedback loop that further sustains leukemia growth and shields the tumor from immune system attacks^[33]^. In addition to driving leukemia progression, the dysregulation of the JAK-STAT pathway in pediatric leukemia can also contribute to chemoresistance. Leukemia cells with constitutively active JAK-STAT signaling may become less responsive to traditional chemotherapy regimens, as the pathway promotes cell survival and prevents apoptosis. By enhancing the expression of anti-apoptotic proteins such as BCL-2 and MCL-1, JAK-STAT signaling can help leukemia cells resist the cytotoxic effects of chemotherapy. Moreover, resistance mechanisms may also be driven by secondary mutations in the JAK-STAT pathway itself or compensatory activation of other pathways, such as the PI3K/AKT or MAPK pathways. These findings underscore the importance of targeting the JAK-STAT pathway as part of a broader therapeutic strategy to overcome chemoresistance and improve patient outcomes^[11]^.

The dysregulation of the JAK-STAT pathway in pediatric leukemia presents a promising target for therapeutic intervention. Targeted therapies that inhibit JAK-STAT signaling, such as JAK2 inhibitors (e.g., ruxolitinib and fedratinib), have shown efficacy in adult hematologic malignancies and are being explored in pediatric settings. These inhibitors block the kinase activity of JAK2, thereby preventing the phosphorylation and activation of STAT proteins and the downstream signaling that drives leukemia progression. Although these therapies are still in early clinical trials for pediatric leukemia, their potential to reduce tumor burden and improve survival rates is promising. Additionally, combining JAK-STAT inhibitors with other therapeutic strategies, such as chemotherapy or immune checkpoint inhibitors, may help to overcome drug resistance and enhance the overall efficacy of treatment^[34]^. Furthermore, the development of biomarker-driven approaches to identify patients with dysregulated JAK-STAT signaling may help to personalize treatment strategies for pediatric leukemia. By using genetic profiling to detect JAK2 mutations, STAT alterations, and other pathway-associated changes, clinicians could tailor therapies to target the underlying molecular drivers of the disease. This precision medicine approach could improve treatment outcomes and reduce the risk of treatment-related toxicity. Additionally, understanding the role of the JAK-STAT pathway in shaping the leukemia microenvironment and influencing immune responses could lead to the development of combination therapies that restore immune surveillance and promote leukemia cell death^[35]^.

### Interaction of JAK-STAT dysregulation with the leukemia microenvironment in pediatric leukemia

The interaction between leukemia cells and the bone marrow microenvironment plays a crucial role in the pathogenesis and progression of pediatric leukemia. The TME is a complex network composed of stromal cells, extracellular matrix components, immune cells, and blood vessels, which collectively provide a supportive niche that sustains leukemia growth and survival. Dysregulated signaling pathways, particularly the JAK-STAT pathway, significantly influence the dynamics of the leukemia microenvironment, creating a mutually beneficial relationship that fosters leukemia cell proliferation, immune evasion, and drug resistance^[31]^. One of the key aspects of JAK-STAT pathway dysregulation in pediatric leukemia is the ability of leukemic cells to modulate the TME to promote their own survival and growth. When JAK2 mutations or aberrant STAT activation occur in leukemia cells, they often lead to the upregulation of pro-inflammatory cytokines such as IL-6, IL-10, TNF-α, and GM-CSF. These cytokines act as signaling molecules that not only support the proliferation and survival of leukemia cells but also influence the activity of various components within the TME. For example, IL-6 is known to activate STAT3, a key transcription factor in leukemia, leading to the promotion of survival pathways, anti-apoptotic proteins (e.g., BCL-2), and pro-angiogenic factors, all of which contribute to the persistence of leukemia cells in the bone marrow. This cytokine-induced activation of the TME can create a feedback loop that perpetuates leukemia progression and resistance to conventional therapies^[32]^.

Furthermore, JAK-STAT pathway activation plays a central role in the recruitment and manipulation of immune cells within the TME, thereby promoting immune evasion. Leukemia cells with dysregulated JAK-STAT signaling can induce the recruitment of Tregs, myeloid-derived suppressor cells (MDSCs), and macrophages, all of which have immunosuppressive properties. These immune cells, once recruited into the TME, contribute to a hostile environment for anti-tumor immune responses. For instance, Tregs suppress T-cell activation and cytokine production, preventing effective anti-leukemia immune responses. Moreover, MDSCs and macrophages can produce factors like TGF-β and IL-10, which further promote immune tolerance and inhibit cytotoxic T-cell function. By promoting the accumulation of these immune-suppressive cells, JAK-STAT signaling allows leukemia cells to evade immune surveillance and continue to thrive in the bone marrow^[33]^. Another significant impact of JAK-STAT pathway dysregulation is the modification of the extracellular matrix (ECM) within the TME. The ECM provides structural support to the bone marrow microenvironment and regulates cell adhesion, migration, and differentiation. Dysregulated JAK-STAT signaling can influence the secretion of ECM components like fibronectin, collagen, and laminin, which not only enhance leukemia cell adhesion to the stromal cells but also facilitate leukemia cell migration and invasion into other tissues. Additionally, the activation of JAK-STAT signaling pathways in stromal cells can increase the expression of angiogenic factors, such as VEGF (vascular endothelial growth factor), which promotes the development of blood vessels within the bone marrow. This neovascularization process creates an enriched vascular niche that further supports the growth and expansion of leukemia cells, offering them a continuous supply of nutrients and oxygen, and enhancing the potential for leukemia cell dissemination to distant sites^[11]^.

In the context of drug resistance, the leukemia microenvironment, influenced by dysregulated JAK-STAT signaling, may contribute to the persistence of leukemia cells following chemotherapy or targeted treatment. In particular, leukemia cells can exploit the TME to shield themselves from the cytotoxic effects of conventional therapies. The upregulation of anti-apoptotic proteins, such as BCL-2 and MCL-1, in response to JAK-STAT activation, can make leukemia cells more resistant to the pro-apoptotic effects of chemotherapeutic agents. Additionally, the cytokines produced by stromal cells and immune cells in the TME can activate survival signaling pathways in leukemia cells, enabling them to resist the cell death induced by chemotherapy. The immune-suppressive nature of the TME, influenced by JAK-STAT signaling, may also limit the efficacy of immunotherapies, including chimeric antigen receptor T-cell (CAR-T) cell therapy and immune checkpoint inhibitors, which rely on the activation of T-cells to kill leukemia cells^[34]^. The dynamic interaction between dysregulated JAK-STAT signaling and the leukemia microenvironment highlights the importance of targeting both leukemia cells and the supportive stromal niche in therapeutic approaches. Strategies aimed at disrupting JAK-STAT signaling in leukemia cells could help to destabilize the supportive network that sustains leukemia growth. Additionally, therapies designed to modify the TME, such as immune checkpoint inhibitors, cytokine blockade, or TME-targeted therapies, may enhance the effectiveness of JAK-STAT inhibitors. For example, combining JAK-STAT inhibitors with immunomodulatory agents may reverse the immune suppression in the TME, restoring anti-leukemia immunity and improving therapeutic outcomes. Furthermore, targeting the ECM components or angiogenic signals that promote leukemia cell survival and dissemination could prevent leukemic cells from exploiting the bone marrow niche for metastatic growth^[35]^.

## Drug development targeting JAK2

### JAK2 inhibitors in pediatric leukemia

JAK2 inhibitors are a class of drugs designed to target the JAK2 protein, a key regulator of the JAK-STAT signaling pathway, which is involved in many cellular processes, including proliferation, survival, and differentiation of hematopoietic cells. Dysregulation of JAK2, particularly due to mutations such as the V617F mutation, is commonly implicated in the development and progression of hematological malignancies, including pediatric leukemia. By targeting JAK2, these inhibitors aim to block the aberrant signaling that drives leukemogenesis, offering a potential therapeutic option for treating JAK2-driven pediatric leukemia^[25,36]^.

#### Mechanisms of JAK2 inhibition

JAK2 is a tyrosine kinase that functions by phosphorylating and activating STAT proteins, particularly STAT5, which are critical in mediating the transcriptional regulation of genes involved in cell survival and proliferation. In many leukemia cases, mutations in JAK2 lead to its constitutive activation, allowing leukemia cells to proliferate uncontrollably. JAK2 inhibitors work by binding to the ATP-binding pocket of JAK2 thereby prevents its activation. This inhibition interrupts the downstream signaling cascades, preventing the activation of STAT proteins and halting the transcription of genes essential for leukemia cell growth and survival. There are two primary classes of JAK2 inhibitors: selective and non-selective. Selective inhibitors target JAK2 specifically, while non-selective inhibitors can also target other JAK family members, such as JAK1, JAK3, and TYK2, which can lead to broader effects on cytokine signaling. For leukemia treatment, selective JAK2 inhibitors are preferred because they provide more targeted inhibition, minimizing the risk of off-target effects on normal hematopoiesis^[37]^.

#### Current JAK2 inhibitors and their clinical applications

The most well-known and widely used JAK2 inhibitor is ruxolitinib, which is FDA-approved for the treatment of myelofibrosis and polycythemia vera in adult populations. Ruxolitinib is a potent, oral JAK1/2 inhibitor that effectively reduces the splenomegaly and symptomatic burden of these diseases. Although ruxolitinib has not been widely tested in pediatric leukemia, preclinical studies and early-phase clinical trials suggest that it holds promise as a treatment for pediatric leukemias with JAK2 mutations or JAK-STAT pathway dysregulation. In these studies, ruxolitinib has been shown to inhibit leukemia cell proliferation and induce apoptosis in JAK2-mutant leukemia models. Another JAK2 inhibitor currently under investigation is fedratinib, which is more selective for JAK2 and has shown efficacy in treating myeloproliferative disorders in adults. Fedratinib has a more potent effect on JAK2 V617F mutations, which are frequently associated with chronic myelomonocytic leukemia and AML. While its primary use has been in adult populations, its potential in pediatric leukemia, particularly those with JAK2 mutations, is being explored in clinical trials. The drug’s ability to selectively inhibit JAK2 activity makes it a promising candidate for targeted therapies in pediatric patients^[38,39]^.

#### Potential role in pediatric leukemia

In pediatric leukemia, JAK2 inhibitors could be particularly valuable for patients with JAK2 mutations or dysregulated JAK-STAT signaling, such as those with ALL, AML, and CML. These mutations often confer resistance to traditional chemotherapy, making JAK2 inhibition a promising strategy to overcome this challenge. By targeting the JAK2 pathway, these inhibitors can restore normal cell signaling, reduce leukemia cell survival, and enhance the sensitivity of leukemia cells to chemotherapy. A key advantage of JAK2 inhibitors in pediatric leukemia is their potential to specifically target leukemia cells while sparing normal hematopoietic cells. The selective inhibition of JAK2 can potentially minimize the risk of cytopenias and other hematologic side effects, which are common in conventional chemotherapy. Furthermore, combination therapies that combine JAK2 inhibitors with chemotherapy, immunotherapy, or epigenetic modulators may enhance the therapeutic effects by targeting multiple mechanisms of leukemia pathogenesis simultaneously^[40,41]^.

### Combination therapies enhancing the efficacy of JAK2 inhibitors in pediatric leukemia

Combination therapies, which involve the use of multiple therapeutic agents, are increasingly being explored to enhance the efficacy of JAK2 inhibitors in the treatment of pediatric leukemia. This approach aims to overcome the challenges of drug resistance, improve treatment outcomes, and reduce adverse effects by targeting multiple pathways involved in the pathogenesis of leukemia. The JAK2-STAT signaling pathway plays a critical role in the survival, proliferation, and differentiation of hematopoietic cells, including leukemia cells and its dysregulation is frequently observed in various types of leukemia. Combining JAK2 inhibitors with other treatment modalities offers the potential to target this pathway while simultaneously addressing other critical mechanisms of leukemogenesis, such as immune evasion, apoptosis resistance, and chemotherapy resistance^[42,43]^.

#### JAK2 inhibitors and chemotherapy combination

One of the primary challenges in treating pediatric leukemia is the development of chemoresistance, where leukemia cells become resistant to traditional chemotherapy drugs. JAK2 inhibitors offer a novel approach to overcome this resistance. Since JAK2 mutations and dysregulated JAK-STAT signaling often contribute to leukemia cell survival, the use of JAK2 inhibitors in combination with conventional chemotherapy can sensitize leukemia cells to chemotherapeutic agents. By blocking the survival signals provided by the JAK2 pathway, these inhibitors may restore chemotherapy sensitivity and enhance apoptosis, leading to improved therapeutic outcomes. For example, in ALL or AML, JAK2 inhibitors such as ruxolitinib can help reduce leukemia cell resistance to chemotherapy by disrupting the aberrant signaling that sustains leukemia cell survival. Combining JAK2 inhibition with chemotherapeutic agents like cytarabine, daunorubicin, or methotrexate may enhance the efficacy of these drugs, making the leukemia cells more vulnerable to treatment. Furthermore, the combination of JAK2 inhibitors and chemotherapy could reduce the chemoresistance-associated side effects, such as myelosuppression and cytopenias, by targeting the leukemia cells more specifically^[44]^.

#### JAK2 inhibitors and immunotherapy combination

Immunotherapy has become a promising treatment modality for various cancers, including leukemia. CAR-T therapy and immune checkpoint inhibitors have shown promising results in hematological malignancies. However, one of the limitations of immunotherapy in pediatric leukemia is the immune evasion mechanism employed by leukemia cells. Dysregulated JAK2-STAT signaling can impair immune surveillance, allowing leukemia cells to evade detection and destruction by the immune system. By combining JAK2 inhibitors with immunotherapeutic strategies, it may be possible to re-sensitize leukemia cells to immune attack and enhance the efficacy of immune-based therapies. For example, combining JAK2 inhibitors with immune checkpoint inhibitors such as anti-PD-1 or anti-CTLA-4 antibodies could enhance the immune response against leukemia cells. The inhibition of the JAK2 pathway can help reverse the immunosuppressive effects within the TME, thereby improving the activity of CAR-T cells or tumor-infiltrating lymphocytes. Additionally, JAK2 inhibition may promote the differentiation of MDSCs and Tregs, which are known to contribute to immune suppression in leukemia, thereby improving the overall immune response^[45,46]^.

#### JAK2 inhibitors and epigenetic modulators combination

Leukemia cells often undergo epigenetic changes that alter gene expression, contributing to leukemogenesis and drug resistance. The combination of JAK2 inhibitors with epigenetic modulators, such as histone deacetylase inhibitors (HDAC inhibitors) or DNA methyltransferase inhibitors (DNMT inhibitors), offers the potential to reverse the epigenetic alterations that contribute to leukemia progression and resistance. Epigenetic dysregulation in pediatric leukemia may affect key genes involved in apoptosis, DNA repair, and cell cycle regulation, making combination therapies with JAK2 inhibitors an attractive strategy. For instance, combining JAK2 inhibitors with HDAC inhibitors like vorinostat or panobinostat may lead to re-expression of tumor suppressor genes and enhance leukemia cell differentiation, thus complementing the effects of JAK2 inhibition. This combination could also help in overcoming drug resistance by reprogramming the epigenetic landscape of leukemia cells, making them more susceptible to JAK2 inhibition and other therapeutic agents^[47,48]^.

#### JAK2 inhibitors and targeted therapies combination

Pediatric leukemia is characterized by the presence of various genetic mutations and aberrant signaling pathways that drive leukemia progression. By combining JAK2 inhibitors with other targeted therapies, such as inhibitors of BCL-2 (to promote apoptosis) or FLT3 inhibitors (to block additional tyrosine kinase signaling), it is possible to simultaneously target multiple signaling pathways that drive leukemia. FLT3 mutations are commonly found in AML, and BCL-2 overexpression is linked to apoptosis resistance. Combining JAK2 inhibitors with BCL-2 inhibitors such as venetoclax could synergistically enhance leukemia cell death by both disrupting survival signaling (via JAK2 inhibition) and promoting apoptosis (via BCL-2 inhibition). Additionally, combining JAK2 inhibitors with IDH inhibitors (targeting isocitrate dehydrogenase mutations in leukemia) or TKIs (such as imatinib in CML) could provide a comprehensive treatment approach that targets both the JAK-STAT pathway and other critical oncogenic drivers in pediatric leukemia. This multi-pronged approach can help overcome single-drug resistance and improve the overall effectiveness of treatment^[49]^.

#### JAK2 inhibitors and conventional therapies combination

Finally, JAK2 inhibitors may be combined with conventional therapies such as stem cell transplantation (HSCT). The combination of JAK2 inhibition and HSCT could be particularly beneficial in cases where JAK2 mutations or JAK-STAT dysregulation contribute to relapse or minimal residual disease after transplantation. By targeting the residual leukemia cells that survive chemotherapy and HSCT, JAK2 inhibitors may improve the outcomes of transplantation and reduce the likelihood of relapse^[49]^.

### The role of JAK2 inhibition in overcoming drug resistance in pediatric leukemia

Drug resistance is one of the most significant challenges in the treatment of pediatric leukemia, often leading to relapse and treatment failure. It can develop through various mechanisms, including genetic mutations, upregulation of survival pathways, and the protective tumor microenvironment. JAK2 inhibitors, as a part of targeted therapy, offer a promising strategy to combat resistance. By targeting the JAK-STAT signaling pathway, which is often dysregulated in leukemic cells, JAK2 inhibitors can overcome multiple layers of drug resistance in leukemia, improving treatment outcomes.

#### Targeting JAK2-driven resistance mechanisms

In pediatric leukemia, JAK2 mutations or dysregulation of the JAK-STAT pathway are frequently implicated in the survival and proliferation of leukemic cells, even in the presence of chemotherapy. These mutations allow leukemia cells to bypass apoptotic signals and continue proliferating, contributing to drug resistance. For instance, JAK2 V617F mutation is associated with a variety of hematologic malignancies and can confer resistance to chemotherapy and other tyrosine kinase inhibitors. In such cases, JAK2 inhibitors like ruxolitinib or fedratinib specifically block the aberrant JAK2 signaling, disrupting the leukemic cells’ ability to evade apoptosis and promoting cell death. Targeting JAK2-mediated pathways can also overcome cross-resistance to other agents. For example, JAK2 inhibitors have been shown to reverse resistance to chemotherapeutic agents such as cytarabine and daunorubicin in AML. By inhibiting JAK2, the resistance mechanisms driven by altered signaling pathways are counteracted, making the leukemia cells more susceptible to conventional therapies. Moreover, JAK2 inhibition can synergize with other drugs to achieve a combinatorial effect that may reduce the likelihood of resistant clones emerging^[50,51]^.

#### Overcoming immune evasion and tumor microenvironment resistance

Leukemia cells often develop mechanisms to evade immune detection, which contributes to their survival despite therapy. The TME, which includes stromal cells, immune cells, and extracellular matrix components, plays a significant role in this immune evasion. JAK2 dysregulation in the TME contributes to immune suppression, fostering an environment in which leukemia cells are protected from the immune system. JAK2 inhibitors can disrupt the immunosuppressive TME, reducing the activity of Tregs and MDSCs, which are key players in immune evasion. Moreover, combining JAK2 inhibitors with immune checkpoint inhibitors, such as anti-PD-1 or anti-CTLA-4, can further overcome immune resistance. By inhibiting JAK2, the immune surveillance system becomes more effective in recognizing and destroying leukemia cells, even in the presence of immunosuppressive factors in the TME. This combination approach not only targets the leukemia cells directly but also reprograms the immune system to recognize and eliminate resistant clones, potentially leading to a more sustained remission^[52,53]^.

#### Reducing resistance to targeted therapies

Leukemia cells often acquire resistance to targeted therapies, such as those targeting specific tyrosine kinases or BCL-2. This resistance can occur through mutation acquisition or by activation of alternative survival pathways. By combining JAK2 inhibitors with other targeted therapies, drug resistance can be circumvented. For example, combining ruxolitinib (a JAK2 inhibitor) with BCL-2 inhibitors, such as venetoclax, has shown promising results in treating AML and ALL by targeting both the anti-apoptotic mechanisms and JAK-STAT pathway. This dual inhibition leads to more efficient leukemic cell death and reduces the likelihood of developing resistance to either drug alone. Additionally, combining JAK2 inhibitors with epigenetic modifiers (e.g., histone deacetylase inhibitors (HDAC inhibitors) or DNA methyltransferase inhibitors) can reprogram the leukemia cell epigenome, making them more susceptible to both JAK2 inhibition and chemotherapeutic agents. Epigenetic changes can often contribute to drug resistance, and by targeting these alterations, JAK2 inhibitors may improve the overall treatment response and overcome resistance to other therapies^[51,54]^.

#### Overcoming relapse through minimal residual disease (MRD) control

Minimal residual disease (MRD) refers to the small number of leukemic cells that remain after treatment and can eventually lead to relapse. MRD can often escape detection by conventional methods but may lead to therapeutic failure if not properly targeted. JAK2 inhibitors can play an essential role in preventing relapse by targeting the residual leukemic cells that persist after chemotherapy. The JAK-STAT pathway is known to be involved in the maintenance of leukemia stem cells, which are often resistant to standard chemotherapy and responsible for disease relapse. By specifically targeting JAK2-driven signaling, inhibitors can reduce the survival and self-renewal capacity of leukemia stem cells, preventing them from contributing to MRD and relapse. Moreover, combining JAK2 inhibitors with allogeneic stem cell transplantation (HSCT) could be a strategy to target MRD while reducing the risk of post-transplant relapse in high-risk pediatric leukemia patients. This combination not only enhances the eradication of leukemic stem cells but also provides a more durable and effective remission^[52]^.

#### Overcoming resistance to epigenetic therapy

Resistance to epigenetic therapy, which targets histone modification and DNA methylation, is also a growing concern in pediatric leukemia treatment. Epigenetic modifications can drive leukemia progression and drug resistance. JAK2 inhibitors can be used to enhance the effectiveness of epigenetic therapies by modifying the epigenetic landscape of leukemia cells. By inhibiting JAK2, which is involved in the regulation of gene expression and cell differentiation, the combination of JAK2 inhibitors with epigenetic modulators (e.g., HDAC inhibitors or DNMT inhibitors) can help restore normal cell differentiation and overcome resistance to epigenetic reprogramming therapies. Furthermore, this approach can also target leukemia cells that are in a quiescent or drug-resistant state, which are often overlooked by conventional treatments. By reprogramming these cells through the combined inhibition of JAK2 and epigenetic pathways, the likelihood of resistance can be reduced, leading to more successful and lasting treatment responses^[53]^.

## Challenges in targeting JAK2 in pediatric leukemia

Despite the promising potential of JAK2 inhibitors in treating pediatric leukemia, several challenges hinder their widespread application and efficacy in clinical practice. These challenges span from the inherent complexities of the disease itself to the limitations of current therapeutic strategies.

### Heterogeneity of pediatric leukemia

Pediatric leukemia encompasses a wide range of subtypes, each with distinct molecular and genetic characteristics. This heterogeneity complicates the development of universal therapies, as JAK2 mutations or JAK-STAT pathway dysregulation may not be present or play a prominent role in all cases of leukemia. For instance, ALL and AML exhibit significant genetic and epigenetic differences, and JAK2 mutations may be more prevalent in some subtypes than others. Thus, targeting JAK2 may not be universally effective across all pediatric leukemia cases, and treatments must be tailored to the specific genetic profile of the leukemia. The identification of biomarkers to predict which patients will benefit from JAK2 inhibitors is a significant challenge that remains to be fully addressed^[55]^.

### Development of drug resistance

While JAK2 inhibitors offer a promising therapeutic option, drug resistance remains a significant challenge. Leukemia cells may develop resistance to JAK2 inhibitors through various mechanisms, such as secondary mutations in JAK2 or other components of the JAK-STAT pathway. These mutations can render the inhibitors less effective or entirely ineffective. Additionally, clonal evolution in leukemia cells can lead to the emergence of resistant subpopulations that continue to proliferate despite treatment. As with many targeted therapies, the plasticity of leukemia cells and their ability to activate alternative signaling pathways further complicates treatment strategies. Overcoming these resistance mechanisms will require the development of next-generation JAK2 inhibitors or combination therapies that can address resistance at multiple levels^[56]^.

### Toxicity and side effects

Like most targeted therapies, JAK2 inhibitors are not without their side effects. The blockade of JAK2 can affect not only leukemic cells but also normal hematopoietic cells, leading to adverse effects on hematopoiesis. Common side effects include anemia, thrombocytopenia, and neutropenia, which can compromise the patient’s ability to fight infections and lead to bleeding complications. Additionally, the use of JAK2 inhibitors can result in gastrointestinal symptoms, fatigue, and liver toxicity, which can limit their long-term use in pediatric patients. The balance between efficacy and toxicity is crucial, especially in pediatric populations, who may have a lower tolerance to side effects due to their developing immune and organ systems. Personalized **dosing** and close monitoring will be essential to minimize these risks^[57]^.

### Tumor microenvironment and immune evasion

The TME plays a critical role in the progression and resistance of pediatric leukemia. Leukemic cells can thrive by creating a supportive environment that shields them from immune surveillance and promotes resistance to treatment. The JAK-STAT pathway is often upregulated in the TME, contributing to immune suppression and providing a growth advantage to leukemia cells. However, targeting JAK2 in isolation may not be sufficient to overcome the complex interplay between leukemia cells and their surrounding environment. The immunosuppressive properties of the TME, including the presence of Tregs and MDSCs, can inhibit the effectiveness of JAK2 inhibitors. Moreover, therapies that target JAK2 could have unintended consequences on the immune system, further complicating the treatment approach^[53]^.

### Limited understanding of long-term effects

Although JAK2 inhibitors are showing promise in preclinical and early-phase clinical trials, there is still a limited understanding of their long-term effects, particularly in pediatric patients. Leukemia in children is a chronic disease, and long-term therapy with JAK2 inhibitors could have unforeseen consequences, including the potential for secondary malignancies or late-onset toxicities. The long-term effects of JAK2 inhibition on normal hematopoiesis and organ function remain under investigation, and careful long-term monitoring is required to assess the safety of these therapies in pediatric populations. Given that childhood leukemia treatments often lead to lifelong health challenges, it is vital to comprehensively evaluate the balance between short-term efficacy and long-term toxicity^[58]^.

### Cost and accessibility

Another significant challenge is the cost and accessibility of JAK2 inhibitors, particularly in low-resource settings. The high **cost** of these targeted therapies may limit their availability, especially in countries or regions where healthcare resources are scarce. In addition, pediatric patients require careful monitoring and dose adjustments, which adds to the complexity and cost of treatment. Ensuring equitable access to JAK2 inhibitors and other emerging therapies is essential for improving outcomes in pediatric leukemia, particularly in underserved populations^[59]^.

## Future directions in targeting JAK2 in pediatric leukemia

The development of JAK2 inhibitors represents a significant advancement in the treatment of pediatric leukemia, but several areas remain for improvement and further exploration. Continued research and innovation are essential to optimize the use of JAK2-targeted therapies, address existing challenges, and improve outcomes for pediatric leukemia patients. Here are some future directions that could guide the field forward:

### Personalized treatment approaches

As pediatric leukemia is highly heterogeneous, a one-size-fits-all approach to treatment may not be effective. Moving forward, there is a growing need for personalized medicine, where treatment regimens are tailored to the unique molecular profile of each patient. Advances in genetic sequencing and biomarker identification can help identify which specific JAK2 mutations or dysregulated pathways are present in a particular patient’s leukemia. This information could enable clinicians to select the most appropriate JAK2 inhibitors or combination therapies based on individual genetic and molecular profiles, thereby increasing treatment efficacy and minimizing unnecessary side effects^[60]^.

### Exploration of combination therapies

Given the complex nature of pediatric leukemia and the potential for drug resistance, combination therapies are likely to play a pivotal role in overcoming the limitations of JAK2 inhibitors. Future research should focus on combining JAK2 inhibitors with other targeted therapies, such as inhibitors of the PI3K/AKT or MAPK pathways, which are frequently activated in leukemia. Additionally, combining JAK2 inhibitors with chemotherapy, immunotherapy, or epigenetic modulators could enhance therapeutic responses and reduce the likelihood of relapse. Investigating the synergy between JAK2 inhibitors and immune checkpoint inhibitors may also offer a promising approach to reinvigorate anti-leukemic immune responses in the tumor microenvironment^[52]^.

### Development of second-generation JAK2 inhibitors

While current JAK2 inhibitors have shown promise, the development of second-generation JAK2 inhibitors is crucial for improving clinical outcomes. These next-generation inhibitors may have greater potency, selectivity, and efficacy in overcoming JAK2 mutations associated with drug resistance. One promising approach could involve the development of allosteric inhibitors that bind to regions of JAK2 beyond the ATP-binding site, making them less susceptible to mutations that confer resistance. Additionally, dual inhibitors that target both JAK2 and other signaling molecules implicated in leukemia, such as FLT3, BCR-ABL, or RAS, may provide a more comprehensive therapeutic approach for patients with relapsed or refractory leukemia^[61]^.

### Targeting the tumor microenvironment

The TME plays a critical role in leukemia progression and resistance to treatment. JAK2 inhibitors may need to be used in combination with therapies targeting the TME to improve treatment efficacy. Future studies could explore how JAK-STAT signaling affects immune cell infiltration, stromal cells, and angiogenesis within the TME. Strategies that block immune checkpoint molecules such as PD-1 or CTLA-4 may also synergize with JAK2 inhibitors, enhancing immune-mediated leukemia elimination. Understanding the reciprocal interactions between leukemia cells and their microenvironment will help identify new therapeutic opportunities for overcoming the immune suppression and drug resistance associated with leukemia^[62]^.

### Pediatric-specific dosing and toxicity profiling

The pharmacokinetics and pharmacodynamics of JAK2 inhibitors in pediatric populations require further investigation, as children metabolize drugs differently from adults. Pediatric-specific dosing regimens are essential to minimize toxicity and ensure optimal therapeutic levels of JAK2 inhibitors. Future research should focus on age-appropriate formulations of these inhibitors, as well as studying the long-term effects on growth, development, and organ function. Comprehensive toxicity profiles for pediatric populations will be critical to guide clinicians in balancing treatment efficacy with the risk of adverse events, particularly hematological toxicities such as neutropenia and thrombocytopenia^[63]^.

### Investigating the role of epigenetics in JAK2 signaling

Epigenetic regulation plays a critical role in the development and progression of leukemia. DNA methylation, histone modification, and non-coding RNA expression are important factors influencing JAK2-mediated signaling pathways in leukemia. Future research should explore the potential for epigenetic modulators in combination with JAK2 inhibitors to alter the leukemia epigenome, potentially restoring normal cell differentiation and enhancing therapeutic responses. Targeting epigenetic regulators that modulate the JAK-STAT pathway could also provide a novel approach for drug-resistant leukemia^[64]^.

### Clinical trials and biomarker validation

The continued success of JAK2 inhibitors in pediatric leukemia will depend on the conduct of large-scale, well-designed clinical trials. These trials should include detailed assessments of treatment efficacy, long-term survival, relapse rates, and quality of life outcomes. In parallel, identifying biomarkers that can predict response to JAK2 inhibitors will be crucial for selecting the right patients for therapy. Potential biomarkers include JAK2 mutation status, phosphorylation levels of key signaling molecules within the JAK-STAT pathway, and immune cell markers. Validating these biomarkers will help optimize treatment strategies and monitor therapeutic response in real time^[65]^.

### International collaborations for global access

Access to JAK2 inhibitors and cutting-edge treatments remains a challenge in resource-limited settings, where pediatric leukemia is often diagnosed at advanced stages. International collaborations between academic institutions, pharmaceutical companies, and healthcare organizations will be essential to address this disparity. Efforts to reduce the cost of JAK2 inhibitors and improve accessibility in low- and middle-income countries are paramount. Initiatives that focus on improving early diagnosis, treatment availability, and education about pediatric leukemia in underserved regions could dramatically improve patient outcomes globally^[66]^.

## Recommendations for advancing JAK2 targeted therapies in pediatric leukemia

While JAK2 inhibitors offer promising therapeutic potential for pediatric leukemia, a number of critical steps must be taken to optimize their use and improve clinical outcomes. Below are several key recommendations for advancing JAK2-targeted therapies in the management of pediatric leukemia:

### Personalized treatment strategies

Given the genetic and molecular heterogeneity of pediatric leukemia, treatment strategies should be personalized based on the specific JAK2 mutations and JAK-STAT pathway dysregulation present in each patient. Comprehensive genetic profiling and the identification of relevant biomarkers can help guide clinicians in selecting the most appropriate JAK2 inhibitors or combination therapies, improving therapeutic efficacy and minimizing unnecessary side effects. Invest in research to identify and validate reliable predictive biomarkers that can help identify which patients are most likely to benefit from JAK2-targeted therapies. Biomarkers such as JAK2 mutation status or phosphorylation levels of key signaling proteins could enable more precise and effective use of JAK2 inhibitors in clinical practice.

### Focus on combination therapies

Investigate combination therapies that pair JAK2 inhibitors with other targeted therapies, such as PI3K/AKT inhibitors, MAPK pathway inhibitors, or chemotherapy agents. Combinations could enhance therapeutic responses, overcome resistance mechanisms, and prevent relapse in pediatric leukemia patients. Combine JAK2 inhibitors with immunotherapeutic agents, including immune checkpoint inhibitors, to enhance anti-leukemic immune responses. These strategies may help address immune evasion mechanisms in the tumor microenvironment and improve long-term outcomes.

### Develop next-generation JAK2 inhibitors

Develop second-generation JAK2 inhibitors with greater potency and specificity, as well as the ability to overcome drug resistance due to secondary mutations. Innovative approaches, such as the development of allosteric inhibitors or dual inhibitors targeting multiple pathways (e.g., JAK2 and FLT3), may offer more effective treatments for leukemia subtypes that are refractory to current therapies. Efforts should be made to improve the pharmacokinetics of JAK2 inhibitors, potentially designing drugs with longer half-lives that require less frequent dosing and have reduced toxicity profiles, especially for pediatric patients.

### Investigate the tumor microenvironment

Future research should focus on understanding how the TME contributes to leukemia progression and resistance to JAK2 inhibitors. Investigating interactions between leukemic cells and stromal cells, as well as immune cell infiltration, could help develop strategies to counteract immune suppression and improve the effectiveness of JAK2-targeted therapies. Targeting the immunosuppressive components of the TME, such as Tregs, MDSCs, and angiogenesis in combination with JAK2 inhibitors, may provide a more comprehensive approach to eliminating leukemia and improving response rates.

### Pediatric-specific pharmacokinetics and dosing

The development of age-appropriate formulations and dosing regimens for JAK2 inhibitors is crucial, as pediatric patients may metabolize drugs differently from adults. Tailoring dosing to the developmental stage of the child and their individual pharmacokinetic profiles will be key to minimizing toxicity and optimizing efficacy. Long-term follow-up studies should be conducted to assess the long-term safety of JAK2 inhibitors in pediatric patients, with a particular focus on potential effects on growth, development, and organ function. This will ensure that these treatments do not cause significant harm over the course of the patient’s life.

### Overcoming drug resistance

Further investigation into the mechanisms of resistance to JAK2 inhibitors is essential for improving clinical outcomes. Research should focus on secondary mutations in JAK2 and other molecules in the JAK-STAT pathway that may contribute to treatment failure. Developing strategies to sensitize resistant leukemic cells, or designing inhibitors that target multiple points in the pathway, could help overcome resistance and improve treatment success. The use of adaptive therapy regimens, which monitor response to treatment and adjust dosing or therapeutic strategies accordingly, may help minimize the development of resistance and maintain long-term control of leukemia.

### Accelerating clinical trials and data sharing

Increased collaboration between research institutions, pharmaceutical companies, and clinical centers is essential to accelerate the development and testing of JAK2 inhibitors in pediatric leukemia. Multinational, multi-center clinical trials will enable larger sample sizes, helping to identify efficacy, safety, and optimal dosing in diverse populations. Open data sharing platforms and the publication of clinical trial results should be encouraged to increase access to findings and foster collaboration across the research community. This will help reduce redundancy in studies and speed up the translation of new findings into clinical practice.

### Enhancing access to treatment

Efforts should be made to ensure equitable access to JAK2-targeted therapies in resource-limited settings, where pediatric leukemia is often diagnosed at advanced stages. Strategies to reduce the cost of JAK2 inhibitors and improve infrastructure for diagnosis and treatment will be crucial for improving global health outcomes. Increasing awareness among healthcare providers about the potential of JAK2-targeted therapies and ensuring that pediatric leukemia is diagnosed early will be key to improving survival rates in low-resource settings.

### Improving clinical support systems

Pediatric leukemia treatment should include a multidisciplinary approach that combines oncologists, hematologists, immunologists, and pharmacologists to develop tailored treatment plans for each patient. Support for families, including counseling and financial assistance, will also improve patient adherence and quality of life during treatment. Pediatric leukemia patients often face significant emotional and psychological challenges. Providing robust psychosocial support to both patients and their families, including mental health services, will be essential for their well-being and successful treatment outcomes.

## Recommendations

To improve the diagnosis, treatment, and outcomes of pediatric leukemia patients with JAK2 mutations, several key strategies should be considered:

### Early screening and molecular diagnostics

Implement routine JAK2 mutation screening in pediatric leukemia, particularly in high-risk subtypes like Ph-like ALL. Utilize next-generation sequencing and PCR-based assays for precise and early mutation detection. Incorporate cytokine profiling and phospho-STAT **assays** to identify dysregulated JAK-STAT signaling.

### Development of more selective JAK2 inhibitors

Focus on next-generation JAK2-specific inhibitors with reduced off-target effects and improved efficacy. Explore allosteric inhibitors that modulate JAK2 function without affecting other JAK kinases. Develop dual inhibitors targeting both JAK2 and cooperative pathways (e.g., PI3K, MAPK) to overcome resistance.

### Personalized and combination therapies

Adopt personalized medicine approaches using genetic profiling to tailor treatment strategies. Investigate the combination of JAK2 inhibitors with chemotherapy, TKIs, and immunotherapies for enhanced efficacy. Conduct clinical trials to determine optimal drug combinations and dosing regimens in pediatric patients.

### Addressing drug resistance mechanisms

Monitor patients for secondary mutations and compensatory pathway activation that lead to JAK2 inhibitor resistance. Explore the use of epigenetic modulators (e.g., HDAC inhibitors) to enhance JAK2 inhibitor sensitivity. Investigate the bone marrow microenvironment’s role in drug resistance and develop strategies to disrupt leukemic cell survival mechanisms.

### Enhancing safety and reducing toxicity

Conduct longitudinal safety studies to assess the impact of JAK2 inhibitors on pediatric patients. Develop nanoparticle-based drug delivery systems to enhance bioavailability and reduce toxicity. Implement regular monitoring of hematologic and immune function in patients undergoing JAK2-targeted therapy.

### Exploring gene-editing strategies

Investigate CRISPR-Cas9 gene editing as a potential therapeutic approach to correct JAK2 mutations in hematopoietic stem cells. Develop ex vivo gene therapy strategies to reduce the need for long-term drug therapy.

### Increasing awareness and research collaboration

Promote multidisciplinary research collaborations between oncologists, geneticists, and pharmacologists. Establish international leukemia registries to collect data on JAK2 mutations, treatment responses, and outcomes. Increase funding and support for clinical trials to accelerate the development of novel JAK2-targeted therapies.

## Conclusion

The role of JAK2 in pediatric leukemia represents a critical area of research with immense therapeutic potential. Dysregulation of the JAK-STAT pathway is a key driver in the pathogenesis of leukemia, and understanding the intricate molecular mechanisms behind JAK2 mutations and their downstream effects is crucial for the development of effective therapies. Although JAK2 inhibitors have demonstrated promising results, challenges such as drug resistance, the complex tumor microenvironment, and the need for pediatric-specific formulations remain. To fully capitalize on the promise of JAK2-targeted therapies, ongoing research should focus on optimizing drug efficacy, overcoming resistance mechanisms, and exploring combination therapies to enhance treatment outcomes. Personalized approaches, driven by genetic profiling and the identification of predictive biomarkers, will enable clinicians to tailor therapies to individual patients, ensuring the best possible outcomes. Additionally, the development of next-generation inhibitors, along with addressing pharmacokinetic and dosing issues in pediatric populations, will be essential for improving therapeutic success.

## Data Availability

None.
